# The crystal structure of Shethna protein II (FeSII) from *Azotobacter vinelandii* suggests a domain swap

**DOI:** 10.1107/S2059798324005928

**Published:** 2024-07-10

**Authors:** Burak V. Kabasakal, Ciaran R. McFarlane, Charles A. R. Cotton, Anna Schmidt, Andrea Kung, Lucas Lieber, James W. Murray

**Affiliations:** a Department of Life Sciences, Imperial College, LondonSW7 2AZ, United Kingdom; b Turkish Accelerator and Radiation Laboratory, Gőlbaşı, 06830Ankara, Türkiye; c Cambrium GmbH, Max-Urich-Strasse 3, 13355Berlin, Germany; d Bioheuris Inc., 1100 Corporate Square Drive, St Louis, MO63132, USA; University of Cambridge, United Kingdom

**Keywords:** Shethna protein II, FeSII protein, *Azotobacter vinelandii*, nitrogen fixation, oxygen protection, structural biology, domain swapping

## Abstract

The *Azotobacter vinelandii* FeSII protein forms an oxygen-resistant complex with the nitrogenase MoFe and Fe proteins. FeSII is an adrenodoxin-type ferredoxin that forms a dimer in solution. Previously, the crystal structure was solved [Schlesier *et al.* (2016), *J. Am. Chem. Soc.***138**, 239–247] with five copies in the asymmetric unit. One copy is a normal adrenodoxin domain that forms a dimer with its crystallographic symmetry mate. The other four copies are in an ‘open’ conformation with a loop flipped out exposing the 2Fe–2S cluster. The open and closed conformations were interpreted as oxidized and reduced, respectively, and the large conformational change in the open configuration allowed binding to nitrogenase. Here, the structure of FeSII was independently solved in the same crystal form. The positioning of the atoms in the unit cell is similar to the earlier report. However, the interpretation of the structure is different. The ‘open’ conformation is interpreted as the product of a crystallization-induced domain swap. The 2Fe–2S cluster is not exposed to solvent, but in the crystal its interacting helix is replaced by the same helix residues from a crystal symmetry mate. The domain swap is complicated, as it is unusual in being in the middle of the protein rather than at a terminus, and it creates arrangements of molecules that can be interpreted in multiple ways. It is also cautioned that crystal structures should be interpreted in terms of the contents of the entire crystal rather than of one asymmetric unit.

## Abstract 2

The *Azotobacter vinelandii* FeSII protein forms an oxygen-resistant complex with the nitrogenase MoFe and Fe proteins.

## 1. Introduction

Nitrogen is an essential component of DNA and proteins. Nitrogen gas makes up 80% of the atmosphere, but it is very unreactive. Fixing this unreactive nitrogen is an essential biological and industrial process. Biological nitrogen fixation is catalysed by the enzyme nitrogenase, which reduces nitrogen gas to ammonia. The reduction of nitrogen is catalysed by the MoFe protein, an (NifDNifK)_2_ tetramer containing molybdenum. The MoFe protein is in turn reduced by the dinitrogenase reductase, the Fe protein, a dimer of NifH. MoFe has two oxygen-sensitive metalloclusters, the P-cluster and the FeMoCo, which contains the Mo atom. The Fe protein has a low-potential 4Fe–4S cluster at the surface bridging the dimer interface. Both nitrogenase components are irreversibly inhibited by oxygen, which reacts with the low-potential iron–sulfur clusters. MoFe has a half-life of around 10 min in air, and the Fe protein has a half-life of around 45 s (Robson, 1979 ▸; Robson & Postgate, 1980 ▸). The MoFe–Fe protein complex is partially protected from oxygen relative to each protein individually (Schlesier *et al.*, 2016 ▸). As well as direct in­activation of the nitrogenase, reactions of oxygen with nitrogenase proteins probably generate damaging reactive oxygen species (Maier & Moshiri, 2000 ▸).

Organisms that fix nitrogen, known as diazotrophs, use several strategies to protect themselves from oxygen. Some are obligate anaerobes or micro-aerobes. Some diazotrophic aerobes, such as *Azotobacter* species, use ‘respiratory protection’, a fast respiratory rate, to consume oxygen. A second mechanism is ‘conformational protection’. It was noticed that a nitrogen-fixing culture of *A. chroococcum* would stop fixing when oxygenated by strong agitation, but when the agitation stopped the culture would resume nitrogen fixation at the same rate as before (Hill *et al.*, 1972 ▸). This result implied that the nitrogenase was temporarily being inactivated by a ‘conformational’ mechanism.

In the 1960s, Shethna and coworkers isolated several non­heme iron proteins from *A. vinelandii* (Shethna *et al.*, 1968 ▸). The second of these proteins became known as the Shethna protein or FeSII. Early work on purifying nitrogenase found a pink protein that associated with nitrogenase and provided some oxygen protection (Kelly *et al.*, 1967 ▸). This pink protein was then identified as the FeSII protein (Bulen & LeComte, 1972 ▸; Haaker & Veeger, 1977 ▸). FeSII is the basis of the conformational protection (Robson, 1979 ▸). It forms a ternary complex with the MoFe and Fe proteins that is catalytically inactive, but from which active nitrogenase can be recovered, with a half-life in air of about an hour (Robson, 1979 ▸). FeSII is not essential for *A. vinelandii* viability, even under nitrogen-fixing conditions, but the nitrogenase was more sensitive to inactivation in an FeSII-knockout mutant (Moshiri *et al.*, 1994 ▸).

FeSII has only been shown to have protective activity in *A. vinelandii* and *A. chroococcum*, although FeSII-like functionality has been proposed for other ferredoxins associated with nitrogenase operons, for example FdxD in *Rhodobacter capsulatus* (Hoffmann *et al.*, 2014 ▸) and a *Gluconacetobacter* ferredoxin (Lery *et al.*, 2010 ▸; Ureta & Nordlund, 2002 ▸). It is possible that the protective function has appeared more than once, or that similar mechanisms exist in other diazotrophs but have not yet been identified.

FeSII is a 13 kDa adrenodoxin-type ferredoxin protein (midpoint potential −262 mV) with a 2Fe–2S cluster that forms a dimer in solution (Moshiri *et al.*, 1995 ▸). Its crystallization was published in 1995 (Moshiri *et al.*, 1995 ▸) in an orthorhombic crystal form, but no structure was solved. Recently, Schlesier *et al.* (2016 ▸) reported a 2.2 Å resolution crystal structure of FeSII in the same orthorhombic crystal form (PDB entry 5ffi). The observed FeSII crystal form has five copies in the asymmetric unit. One of them is in a ‘closed conformation’, forming a ‘closed’ dimer with a crystallo­graphic symmetry mate. The closed conformation is similar to other adrenodoxin proteins, such as the monomeric *Escherichia coli* Fdx (Kakuta *et al.*, 2001 ▸). The other four copies in the asymmetric unit are in an ‘open’ from. In the open form, the so-called N-loop consisting of residues 59–96 is flipped out from the structure so that the 2Fe–2S cluster is revealed. Specifically, the ‘lid helix’ from residues 68 to 78 is moved away from the cluster. The four open FeSII molecules were interpreted as two dimers of the same type as the closed form, but with the N-loop flipped out. Schlesier and coworkers proposed that this conformational change is triggered by oxidation of the cluster, and the change enables FeSII to form the protective ternary complex with the MoFe and Fe proteins. Small shifts in the elution time from size-exclusion chromatography in different oxidation states were also consistent with a conformational change. However, the open form of FeSII has not been observed in crystals of any other adrenodoxin-type domains.

Protein domain swapping is when two protein molecules exchange equivalent regions in their structures (Liu & Eisenberg, 2002 ▸; Wodak *et al.*, 2015 ▸). In the simplest form, two adjacent monomers swap a region to form a domain-swapped dimer. Some proteins have evolved natural domain swaps relative to an unswapped ancestor (Cahyono *et al.*, 2020 ▸). In other cases the swap is functional or may be involved in disease (Rousseau *et al.*, 2012 ▸). Domain swapping may also be induced by chemical treatment (Cahyono *et al.*, 2020 ▸) or crystallization. For example, in a crystallized PAS domain one of the six noncrystallographic symmetry copies had a domain-swapped C-terminal α-helix (Emami *et al.*, 2009 ▸). In cyanobacterial Psb29 (Bečková *et al.*, 2017 ▸) only one of three crystal forms had a domain-swapped N-terminal α-helix (Bečková *et al.*, 2017 ▸). In these cases, the domain-swapped structure may be regarded as a crystallization artefact and the swapped structure is not biologically relevant. In most domain-swapped structures the swap is of one end of the structure containing the N- and C-terminus. Swaps of internal regions are rarer, but have been observed, such as in the structure of IX/X-binding protein from snake venom (Mizuno *et al.*, 1997 ▸), which has a loop-exchange heterodimer relative to the homologous rat mannose-binding protein (Weis *et al.*, 1991 ▸).

We have independently solved the structure of the FeSII protein in the same crystal form as previously but to a higher resolution. The atoms are in similar positions to the earlier structure, but our interpretation of the open form of the protein is very different. We believe that the open form is caused by a crystallization-induced domain swap. Here, we present evidence that the ‘open’ conformation of FeSII in the crystal structure is best interpreted as a domain-swapped structure.

## 2. Materials and methods

### 2.1. Overexpression and purification of FeSII

A synthetic gene coding for the FeSII from *A. vinelandii* (AVIN_39700) was synthesized by DNA 2.0 (California, USA) and subcloned into a pRSET-A vector (Invitrogen, Carlsbad, California, USA) with no tag. The FeSII gene was PCR-amplified with primers FESII_FOR, 5′-TATTACATATGGCGACGATCTATTTCAGCAGC-3′, and FESII_REV, 5′-ATTATCTCGAGTTATCAGGCGCCACCCG-3′ (restriction sites are underlined), and the product was digested with NdeI and XhoI, and then ligated into vector cut with NdeI and XhoI restriction enzymes.

FeSII protein was produced by transformation of the vector into chemically competent *E. coli* KRX cells (Promega, Wisconsin, USA) and cultivation in 1 l Terrific Broth medium supplemented with 100 µg l^−1^ ampicillin. Expression was induced at an OD_600_ of 0.6–0.8 with 1 g l^−1^l-rhamnose monohydrate. After overnight induction at 18°C, the cells were harvested by centrifugation and resuspended in 10 m*M* HEPES pH 7.4, 1 m*M* MgCl_2_. Cell disruption was performed by sonication. FeSII was purified using a modification of a published method (Moshiri *et al.*, 1995 ▸). 0.4%(*w*/*v*) NaCl and 11%(*w*/*v*) PEG 8000 were added as powder to the lysate. They were mixed gently for 45 min at 4°C and the cell debris was separated by centrifugation. The red supernatant was filtered through a 0.2 µm filter and purified by anion-exchange chromatography on a Q Sepharose column equilibrated with 10 m*M* HEPES pH 7.4, 1 m*M* MgCl_2_. FeSII was eluted using a linear gradient of MgCl_2_ from 0 to 50 m*M* over ten column volumes. The fractions corresponding to the FeSII protein were pooled and concentrated, and further purified by size-exclusion chromatography (HiLoad 16/600 Superdex 200 pg column) in 10 m*M* HEPES pH 7.4, 5 m*M* MgCl_2_, 100 m*M* NaCl.

### 2.2. Structure determination of FeSII

FeSII was crystallized using hanging-drop vapour diffusion with an equal mixture of 0.1 *M* HEPES pH 8.0, 0.2 *M* sodium citrate, 24%(*w*/*v*) PEG 3350, 2%(*v*/*v*) 1-butanol and 40 mg ml^−1^ FeSII. The crystals were cryoprotected for 30 s in the mother-liquor solution with 30%(*v*/*v*) PEG 400 added and were flash-cooled in liquid nitrogen. X-ray diffraction data were collected at 100 K on beamline I03 at Diamond Light Source, UK. For phase determination, a data set was collected at the Fe *K* edge at a wavelength of 1.734 Å. The data were processed and scaled with *xia*2 (Winter, 2010 ▸) and *XDS* (Kabsch, 2010 ▸). The structure was phased with Fe-SAD using the intrinsic Fe atoms of the protein using *AutoSol* (Terwilliger *et al.*, 2009 ▸) from the *Phenix* package (Adams *et al.*, 2010 ▸; Liebschner *et al.*, 2019 ▸). The phases were improved by density modification in *DM* (Cowtan, 2010 ▸) and a preliminary model was built with *Buccaneer* (Cowtan, 2006 ▸). The model was rebuilt manually in *Coot* (Emsley *et al.*, 2010 ▸), cycling with refinement in *phenix.refine* (Afonine *et al.*, 2012 ▸). This model was then refined and rebuilt against a higher resolution native data set at 1.65 Å. The structure was validated with *MolProbity* (Williams *et al.*, 2018 ▸). The final data-collection and refinement statistics are given in Table 1 ▸. Dimer surface areas and interaction energies were predicted with *PISA* (Krissinel, 2010 ▸; Krissinel & Henrick, 2007 ▸). Molecular figures were produced in *PyMOL* (DeLano, 2002 ▸).

## 3. Results

### 3.1. FeSII structure

We independently solved the FeSII structure using Fe-SAD to a final resolution of 1.65 Å in the same orthorhombic *P*2_1_2_1_2 crystal form as observed in 1995 (Moshiri *et al.*, 1995 ▸) and 2016 (Schlesier *et al.*, 2016 ▸). Data-collection and refinement information is given in Table 1 ▸. The asymmetric unit contains five copies of FeSII. One is in the closed conformation similar to other adrenodoxins. The other four are in the open conformation. Although the crystal form is the same, we have chosen symmetry positions of the chains so that the asymmetric unit contains both of the types of dimer that we describe.

The structures are similar, with r.m.s.d.s of 1.1 Å over the closed chain between this work and Schlesier *et al.* (2016 ▸) and of 0.4 Å between the best-matching open chains. The closed chain is more complete than before, with a gap from residues 82 to 92 in the N-loop. In the open chains the N-loop is ordered, and the entire chain is visible, apart from the N- and C-termini.

### 3.2. Interpretation of the crystal structure

**Figure 1:**
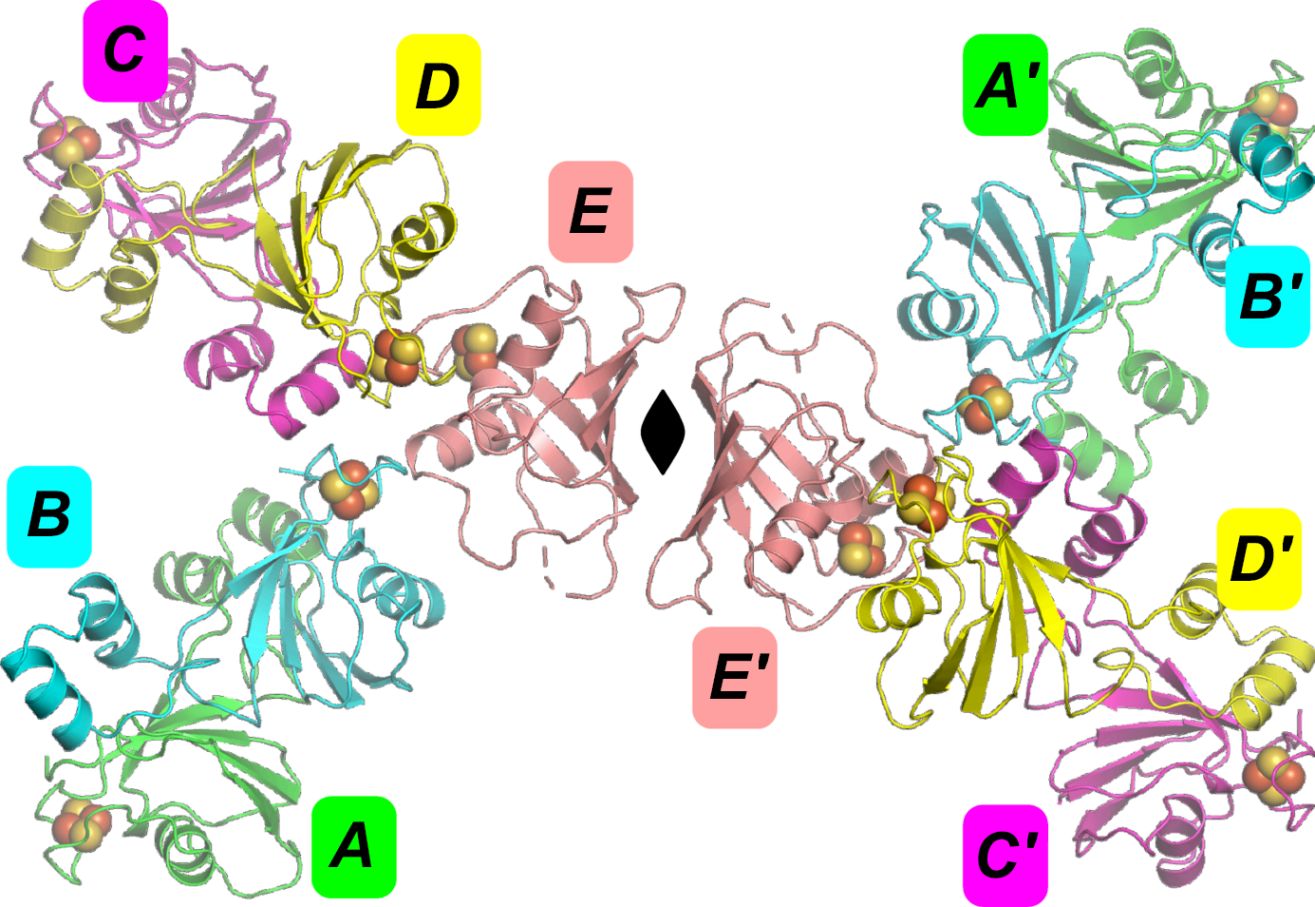
The contents of two unit cells of the FeSII protein, showing the five unique chains *A*, *B*, *C*, *D*, *E* and *A*′, *B*′, *C*′, *D*′, *E*′ for the unit cell next to chain *E*.

In this crystal form the ‘closed’ chain *E* forms a dimer with its crystallographic symmetry mate *E*′ (−*x* + 1, −*y*, *z*). All four open chains form the ‘open dimer’. Chains *A* and *B* form this dimer. Chain *C* forms this dimer with chain *D* from the *x*, *y*, *z* − 1 symmetry mate (chain *D*′). Chains *C* and *D* form another extended interface forming what we call the ‘domain-swap’ dimer. Chain *A* also forms this domain-swap dimer with the *B* chain related by *x*, *y*, *z* − 1 (chain *B*′). All open chains are involved in both the open and domain-swap dimers. Fig. 1 ▸ shows the dimers in the crystal and the chains forming them for two unit cells.

**Figure 2:**
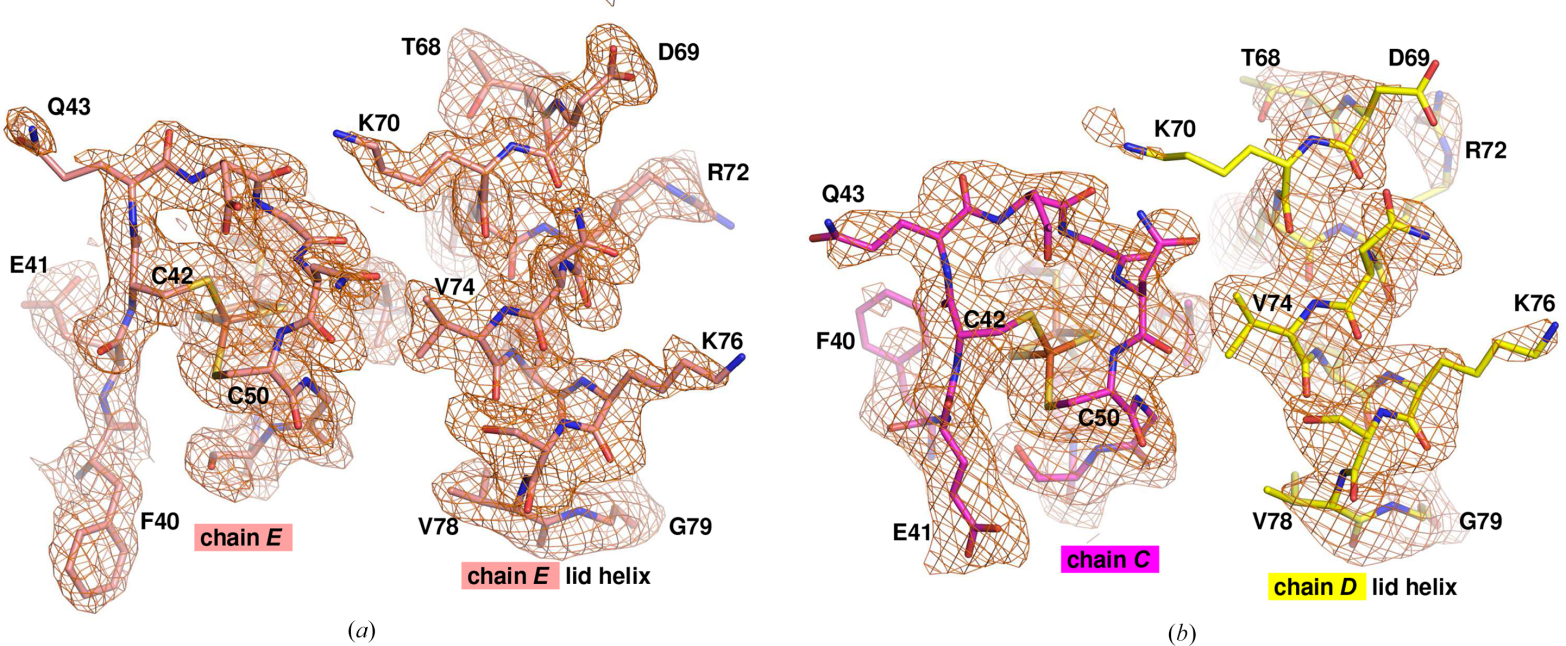
The region around the 2Fe–2S cluster for closed and open chains. In chain *E* (salmon) in the crystal, the lid helix from chain *E* covers the iron–sulfur cluster. In chain *C* (magenta) the lid helix is flipped out in the open conformation, but the cluster is still capped by the lid helix from chain *D*. Weighted 2*F*_o_ − *F*_c_ density was calculated from the 1.65 Å resolution data set and contoured at 0.9σ; selected residues are labelled.

The work by Schlesier and coworkers proposed that the ‘lid helix’ moves away from the 2Fe–2S cluster in the open dimer, exposing it to solvent. However, in the crystal the lid helix in each open chain is replaced by a lid helix from a symmetry-related chain. Fig. 2 ▸(*a*) shows the lid helix in place for chain *E*, the closed chain. For the open chain *C*, Fig. 2 ▸(*b*) shows the lid helix in an equivalent position, but coming from the domain-swap dimer partner chain *D* in the crystal. In chains *A *and *B,* the lid helix is similarly replaced by symmetry-related chains *B*′ and *A*′, respectively.

The predicted interface areas, free energies of dissociation and binding energies for the dimer interfaces that exist in the crystal are shown in Table 2 ▸. The calculated area of the closed dimer interface is 490 Å^2^.

In the open conformation, the ‘lid helix’ (residues 68–79), as part of the longer ‘N-loop’ (residues 59–96), is folded away from the central domain. It forms additional contacts with the closed dimer partner around Pro12, expanding the closed dimer interface. The calculated area of the closed dimer interface is 490 Å^2^, but the mean open dimer interface area is increased by 1120 Å^2^. In the crystal, the open dimers are also involved in the domain-swap dimer. This dimer interface is primarily made up of residues of the opened-out N-loop, but has a mean area of 970 Å^2^, and is the most energetically favoured of the interfaces. The open and domain-swapped dimers have slightly positive predicted free energies of dissociation (Δ*G*^diss^) ranging from 6.2 to 6.7 kcal mol^−1^, suggesting a stable dimer. The closed dimer has a predicted Δ*G*^diss^ of −5.8 kcal mol^−1^, implying a marginally stable dimer within the error of the prediction. These predicted energies suggest that the formation of the open and domain-swap interfaces together is energetically favoured in the crystal.

**Figure 3:**
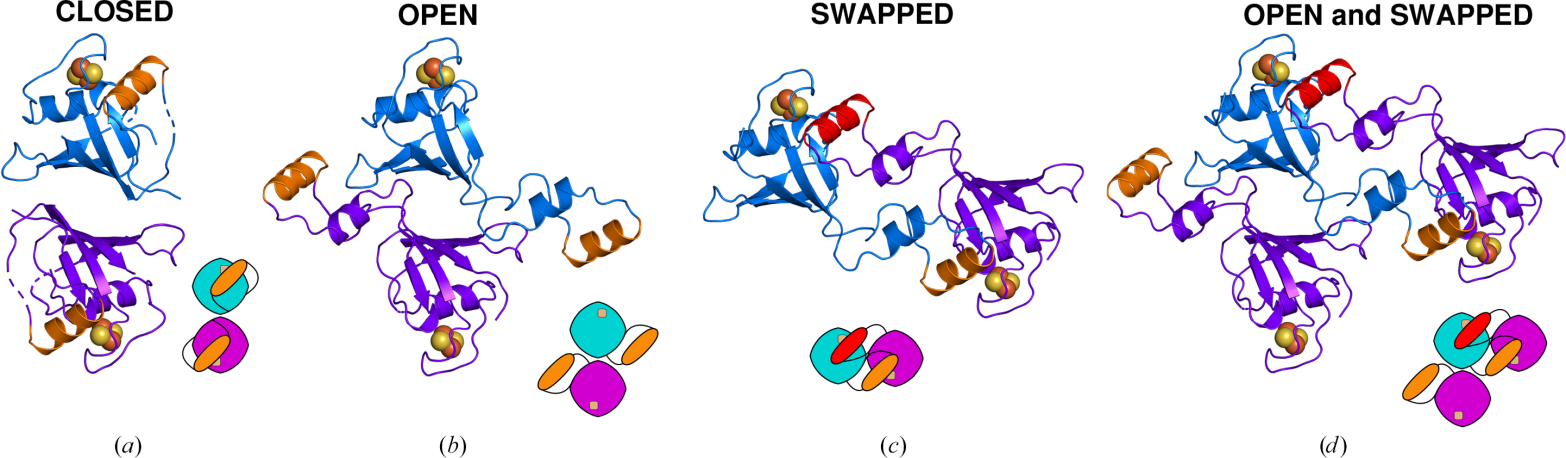
Cartoon and schematic view of the dimers of FeSII in the *P*2_1_2_1_2 crystal form. (*a*) Closed dimer (chains *E* and *E*′). (*b*) Open dimer (chains *A* and *B*). (*c*) Domain-swap dimer (chains *A* and *B*′). (*d*) Open and domain-swap dimers (*A*, *B* and *B*′). The chains are shown in blue and purple, with the lid helix (residues 68–79) in orange, except for the top lid helix when interpreted as part of the domain-swap dimer, when it is shown in red.

The dimers in the FeSII crystal are shown in Fig. 3 ▸ in cartoon and schematic views. The replacement of the lid helix by the lid helix from a symmetry mate (red in Fig. 3 ▸) may be interpreted as a domain swap of the N-loop. This domain swap is unusual in that it involves an internal region of the protein rather than an N- or C-terminus. The swap is also unusual in that it creates an extensive new dimer interface, in addition to the dimer interface that is present in the open and closed dimers. Given that these two interfaces can only occur together in the crystal, we suggest that this open conformation of FeSII is induced by crystallization. The flexibility of the ‘hinge’ regions proposed earlier may allow this protein to change shape under the constraints of crystal lattice formation to a more energetically favoured conformation that may not be biologically relevant.

Schlesier and coworkers proposed that the formation of the open dimers is induced by the oxidation of the 2Fe–2S cluster. Our protein and crystals were prepared aerobically, and our protein has an absorption spectrum corresponding to oxidized protein (Supplementary Fig. S1) so even the closed chain should be oxidized, and thus any conformational change is not driven by differential oxidation states of the 2Fe–2S cluster.

## 4. Discussion

Previous research has claimed that the FeSII protein adopts a radically changed conformation in the oxidized state, based on the crystal structure and small shifts in retention time in size-exclusion chromatography. We have independently solved the FeSII crystal structure at 1.65 Å resolution. Based on examination of the full unit cell of the crystal, we conclude that the ‘open’ structure seen in the crystal is most likely due to crystallization-induced domain-swapping. Previous work on the homologous ferredoxin VI from *Rhodobacter capsulatus* (Sainz *et al.*, 2006 ▸) solved structures of the oxidized and the reduced protein (Armengaud *et al.*, 2001 ▸; Sainz *et al.*, 2006 ▸) and only small conformational changes were seen, mainly around the iron–sulfur cluster. Based on the behaviour of all other known ferredoxins that function without a large conformational change, it is probable that FeSII can also regulate its interaction with nitrogenase with only small conformational changes. We await structure determination of the full MoFe–Fe–FeSII protein complex for a definitive answer to this question.

## Supplementary Material

PDB reference: FeSII from *Azotobacter vinelandii*, 6yav

Supplementary Figure S1. DOI: 10.1107/S2059798324005928/rr5241sup1.pdf

## Figures and Tables

**Table 1:** Data-collection and refinement statistics for FeSII (PDB entry 6yav) Values in parentheses are for the highest resolution shell.

Beamline	I03, Diamond Light Source
Wavelength	0.9763
Data-collection temperature (K)	100
Resolution range (Å)	37.3–1.65 (1.709–1.650)
Space group	*P*2_1_2_1_2
*a*, *b*, *c* (Å)	134.24, 135.1, 37.09
α, β, γ (°)	90, 90, 90
Total reflections	536342 (50652)
Unique reflections	81089 (7901)
Multiplicity	6.6 (6.4)
Completeness (%)	98.33 (97.17)
Mean *I*/σ(*I*)	17.46 (1.47)
Wilson *B* factor (Å^2^)	32.31
*R*_merge_	0.04731 (1.206)
*R*_meas_	0.0515 (1.313)
*R*_p.i.m._	0.02007 (0.513)
CC_1/2_	0.999 (0.568)
CC*	1 (0.851)
Reflections used in refinement	81085 (7901)
Reflections used for *R*_free_	4037 (380)
*R*_work_	0.1979 (0.3162)
*R*_free_	0.2249 (0.3187)
CC(work)	0.966 (0.754)
CC(free)	0.960 (0.759)
No. of non-H atoms	
Total	4656
Macromolecules	4427
Ligands	33
Solvent	196
No. of protein residues	590
R.m.s.d., bond lengths (Å)	0.008
R.m.s.d., angles (°)	1.19
Ramachandran favoured (%)	98.60
Ramachandran allowed (%)	1.40
Ramachandran outliers (%)	0.00
Rotamer outliers (%)	0.00
Clashscore	2.46
Average *B* factor (Å^2^)	
Overall	53.36
Macromolecules	53.66
Ligands	47.41
Solvent	47.75
No. of TLS groups	32

**Table 2:** Surface-area and energy predictions for the *A. vinelandii* FeSII crystal form Interface areas in Å^2^ are calculated as half of the difference in the total accessible surface areas of the isolated and interfacing structures.

Interface	Chains	Interface area (Å^2^)	Δ*G*^diss^ (kcal mol^−1^)	Binding energy Δ*G* (kcal mol^−1^)
Open 1	*A*–*B*	1170	6.2	−9.3
Open 2	*C*–*D*′ (*x*, *y*, *z* − 1)	1080	6.4	−8.4
Mean open	*A*–*B*, *C*–*D*′	1120	6.3	−8.9
Domain swap 1	*C*–*D*	983	6.2	−16.6
Domain swap 2	*A*–*B*′ (*x*, *y*, *z* − 1)	960	6.7	−17.0
Mean domain swap	*C*–*D*, *A*–*B*′	970	6.5	−16.8
Closed	*E*–*E*′ (−*x* + 1, −*y*, *z*)	490	−5.8	−4.8
