# Supplementary material for: The crystal structure of Shethna protein II (FeSII) from Azotobacter vinelandii suggests a domain swap
Source: Acta Crystallogr D Struct Biol. 2024 Jul 10;80(Pt 8):599–604. doi: 10.1107/S2059798324005928 (PMC11301756; doi:10.1107/S2059798324005928)
Supplement: Supplementary file 1 [file d-80-00599-sup1.pdf]

Acta Cryst D STRUCTURAL BIOLOGY

Volume 80 (2024)

Supporting information for article:

The crystal structure of Shethna protein II (FeSII) from *Azotobacter vinelandii* suggests a domain swap

Burak V. Kabasakal, Ciaran R. McFarlane, Charles A. R. Cotton, Anna Schmidt, Andrea Kung, Lucas Lieber and James W. Murray

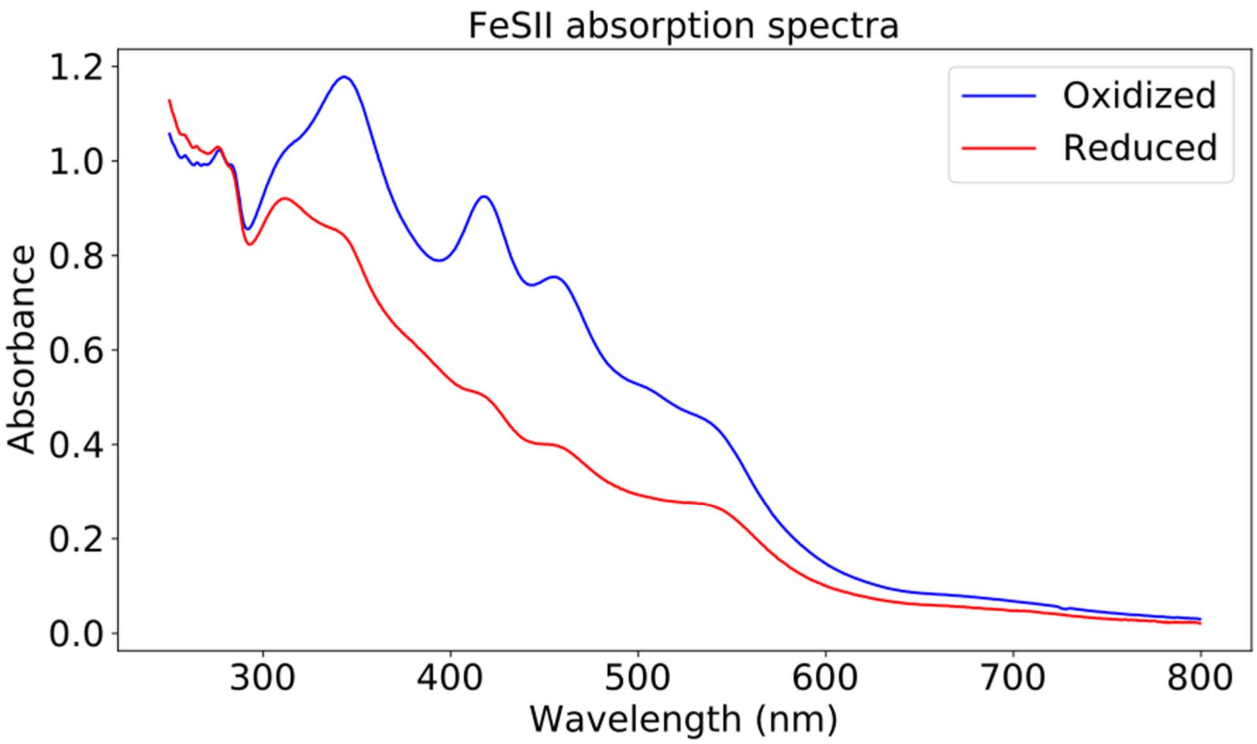

Figure S1 UV-VIS Absorption spectrum of FeSII (1.35 mg/ml) oxidised (blue) and reduced (red) 100 mM HEPES pHh 7.4, 5 mM MgCl2, 100 mM NaCl pH 7.4. Oxidised protein was FeSII prepared in air, reduced protein was prepared by treating anaerobically with 100 µM mM final concentration sodium dithionite, with excess dithionite removed by repeated centrifugal concentration with a 10 kDa cutoff membrane.
